# Examining the impact of leadership practices on nurses’ career competencies: the role of strategic flexibility as a mediator

**DOI:** 10.1186/s12912-025-02890-8

**Published:** 2025-04-30

**Authors:** Amal Diab Ghanem Atalla, Wafaa Hassan Mostafa, Ebaa M. Felemban, Ruba M. Alharazi, Ohood Felemban, Samia Mohamed Sobhi Mohamed

**Affiliations:** 1 Nursing Administration Department, Faculty of Nursing, Alexandria University, Alexandria, Egypt; 2 Nursing Administration Department, Faculty of Nursing, Damanhour University, El-Behira, Egypt; 3 Nursing Education, Public Health Nursing Department, Faculty of Nursing, King Abdulaziz University, Jeddah, Saudi Arabia; 4 Medical Surgical Nursing Department, Faculty of Nursing, King Abdulaziz University, Jeddah, Saudi Arabia; 5 Public Health Nursing Department, Faculty of Nursing, King Abdulaziz University, Jeddah, Kingdom of Saudi Arabia

**Keywords:** Leadership practices, Nurses, Career competencies, Strategic flexibility, Mediating factor

## Abstract

**Background:**

The expansion of nurses’ career competencies and their capacity to adjust to changing healthcare needs are significantly influenced by effective leadership. Strategic flexibility helps nurses improve patient care outcomes, advance professional development, and acquire critical skills by creating a dynamic and responsive work environment.

**Aim:**

To investigate the association between leadership practices and nurses’ career competencies and strategic flexibility’s role as an intermediary in this relationship.

**Design:**

A descriptive cross-sectional study design was used, adhering to STROBE principles.

**Methods and tools:**

Information was gathered from 400 nurses who worked in an Egyptian general hospital namely; El-Rahmania Hospital through structured questionnaires: the multifactor leadership questionnaire, the career competencies questionnaire, and the strategic flexibility questionnaire.

**Results:**

Interpreting the discoveries of this study, a significant direct effect (Estimate = 0.089, *p* = 0.026) between leadership behaviors and strategic flexibility. This suggests that strategic flexibility is positively influenced by leadership. Moreover, leadership behaviors have a significant direct influence on career competencies (Estimate = 0.128, *p* < 0.001), indicating that effective leadership directly enhances career competencies. Furthermore, A significant indirect effect (Estimate = 0.142, *p* = 0.002) is found in the relationship between strategic flexibility and career competencies, indicating that enhancing strategic flexibility enhances the impact of leadership on career development.

**Conclusion:**

The statistically significant relationships between leadership practices, the nurses’ career competencies, and strategic flexibility show how interconnected they are. According to the findings, strategic flexibility significantly mediates the connection between competent nursing practice and effective leadership.

**Nursing implications:**

The study’s conclusions have important ramifications for healthcare policy, leadership development, and nursing practice. Effective leadership techniques that encourage strategic adaptability can equip nurses with the knowledge and abilities needed to successfully negotiate challenging and quickly evolving healthcare contexts. Nurse leaders may increase job satisfaction, lower burnout, and boost retention rates by encouraging resilience, problem-solving skills, and ongoing learning. Additionally, healthcare organizations may guarantee better patient care, more efficiency, and a more engaged nursing workforce by placing a strong priority on strategic flexibility and leadership training.

## Background

Nowadays, the complicated and constantly changing healthcare environment, as well as the shifting demands placed on nurses and the increasing requirements of patients, present enormous challenges for the nursing profession. These difficulties raise the awareness of competent leadership practices value in nursing, which is critical for enhancing a positive work atmosphere and navigating the complexities of healthcare systems [1].

The study is consistent with Resource-Based Theory (RBT) (Barney, 1991). RBT highlights that valuable, uncommon, unique, and non-replaceable resources including human capital—are the source of an organization’s long-term competitive advantage. The model’s leadership practices serve as a means of fostering and advancing nurses’ professional competencies, establishing them as vital assets for the success of the company. This is enhanced by strategic flexibility, which allows leaders to adjust resource allocation to shifting conditions, guaranteeing the ongoing advancement and best use of nursing competencies. By using RBT, the study emphasizes how crucial it is to fund nursing leadership and career advancement as a calculated move to improve the general effectiveness and flexibility of healthcare institutions [2].

The Strategic Directions for Nursing and Midwifery (SDNM) offers evidence-founded procedures together with a connected list of policy primacies that support creating efficient leadership practices, improving nurses’ career competencies, and increasing strategic flexibility [3]. The World Health Organization (WHO), has a significant influence on international health policy and encourages financing for nurse leadership and education. By strengthening the nursing profession and developing critical career competencies, this report aims to progress healthcare admission and fortify nursing worldwide [4]. To improve access to healthcare worldwide and influence universal health policies, this study highlights the importance of investing more in nurse leadership roles. By giving nurses the tools they need to make decisions, think critically, and adapt, strengthening nurse leadership promotes improvements in the field. These skills allow nurses to effectively advocate for equitable healthcare, address global health issues, and help develop creative solutions. This study supports the larger objective of increasing healthcare access and raising the standard and sustainability of care globally by empowering the nursing workforce [5].

## Literature review

### Leadership practices

In addition to maximizing employee creativity, career competencies, and caregiving behaviors, healthcare firms rely on leadership techniques to create a healthy work environment [6]. The phrase “leadership practice” refers to a combination of a leader’s abilities, expertise, and conduct that define how well they collaborate with their team [7]. Examples of leadership practices include the five transformational scales, the three transactional scales, the one laissez-faire scale, and the three result scales. The first transformative scale is called Inspirational Motivation, which describes how a leader articulates and represents a vision. As a result, followers are inspired to look forward to the future with optimism. Idealized Influence (attributed) is the term used to describe the attribution of charisma to a leader. Strong emotional ties, confidence, and trust are all made possible by a leader’s positive attributes. Following one’s ideas and a common purpose is highly valued in idealized influence behavior. Therefore, intellectual stimulation involves challenging followers’ assumptions, how they understand the problems they face and the solutions they devise. Last but not least, individualized consideration consists of taking into account each follower’s unique needs and maximizing their strengths. Contingent reward is a leadership behavior that falls on the transactional leadership scale. It involves a leader concentrating on tasks that are well-defined and rewarding followers after these tasks are accomplished. The leader actively monitors and searches for nonconformities from rules and regulations to prevent them, and corrective actions are subsequently put into place under Active management by exception.

On the other hand, passive intervention only takes place in management by exception when standards are not met or faults have been identified. Laissez-faire, or the lack of leadership, is an even more submissive non-leadership strategy. Lastly, the three outcome criteria are followers’ pleasure with their specific leader, followers’ additional effort, and the usefulness of the leader’s conduct [8]. To improve organizational performance and goal attainment, leadership strategies center on how they behave and interact with nurses [9]. Additionally, it positively affects nurses’ work attitudes, engagement, dedication, and exceptional performance. Furthermore, management support provided by leadership practices fosters nurses’ innovative behaviors and inventiveness, which advances their careers [10]. Additionally, giving them expanding chances to develop their professional skills.

### Career competencies

The roles of nurses have grown as a result of dynamically shifting healthcare environments. Professional growth for nurses depends on their capacity to manage their careers [11]. “professional competencies are the essential knowledge, skills, and attitudes for professional development that an individual can influence and cultivate to function effectively in the business.

There are three primary components to career competencies.: The first is reflective career competencies, which emphasize integrating personal thoughts with one’s professional career and developing an understanding of one’s long-term career. The two career competencies that emerge from this dimension are reflection on motivation and reflection on attributes. They are described as “reflecting on strengths, shortcomings, and skills about one’s career” and “reflecting on values, passions, and motivations about one’s career,” respectively. The capacity to effectively interact with important individuals is referred to as communicative professional competencies, and it is used to increase one’s likelihood of completing tasks. Networking, which is defined as “the awareness of the presence and professional value of an individual network, and the ability to expand this network for career-related purposes,” and self-profiling, which is defined as “presenting and communicating personal knowledge, abilities, and skills to the internal and external labor market,” are the two communicative career competencies that arise from this dimension.

The ability to actively shape one’s career through action is the main focus of behavioral competencies. Career exploration is defined as “actively exploring and searching for work-related and career-related opportunities on the internal and external labor market,” and career control, is defined as “actively influencing learning processes and work processes related to one’s career by setting goals and planning how to fulfill them,” are the two career competencies that stem from this dimension [12, 13]. To support career success and the achievement of work objectives, career competencies can optimize nurses’ knowledge, skills, and attitudes that are necessary for career growth. They help organizations adjust to shifting conditions in addition to enabling nurses to improve their career accomplishments and career self-management. Therefore, career competencies are tools that help nurses manage their careers and improve organizational capabilities like strategic flexibility, which is a key capability that precedes nurses’ competencies by encouraging strategic thinking, offering strategic options, and positively affecting nursing staff members’ skills and professionalism [14, 15].

## Strategic flexibility

The success of an organization is largely dependent on its resources and capabilities as well as how these are used to build other capabilities. In unpredictable and constantly shifting circumstances, strategic flexibility is considered a critical organizational asset for boosting innovation, competencies, and organizational performance [16]. It refers to all of the administrative capacities associated with the organization’s goals or environmental factors and is described as the capacity of the organization to manage changing and turbulent situations [17].

Four dimensions namely: capacity, information, resources, and coordination flexibility can be used to describe strategic flexibility. The ability of the organization to efficiently utilize additional resources and to reallocate services in response to variations in demand is reflected in capacity flexibility. Data must be accessible in a flexible way to be utilized efficiently in the supervisory process. Information flexibility is the capacity of the data to adjust to several applications and users. The ability to manage all of an organization’s resources financial, human, knowledge, and skills allows it to activate its strategic options through various administrative systems. Coordination flexibility is the ability to connect disparate organizational components, enabling compatibility and integration across levels to accomplish shared objectives effectively and efficiently [18, 19].

One instrument that enhances strategy and operational excellence is strategic flexibility. It makes it possible for organizational procedures, knowledge, abilities, and talents to be continuously improved. According to this viewpoint, nursing staff competencies and capacities can be improved, integrated, and developed through strategic flexibility, permanently updating their skill set [20].

### Conceptual model of the study

**Fig. 1:**
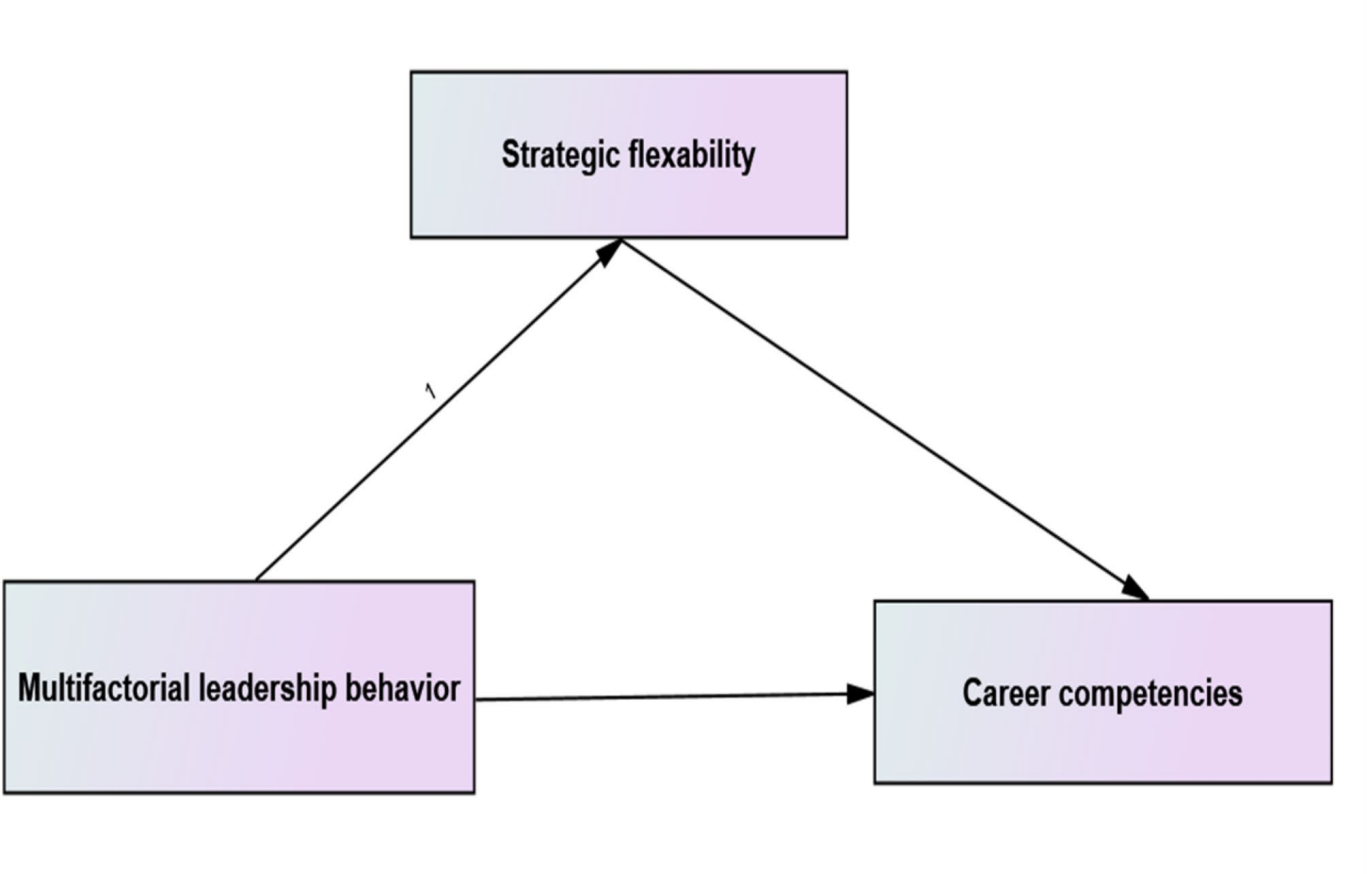
The researchers’ proposed conceptual framework of the study

From the previous conceptualization, for this investigation, the researchers suggested a conceptual model (Fig. 1). Multifactorial leadership behavior is the independent variable, Career Competencies are the dependent variable and strategic flexibility plays a mediating role.

### Significance of the study

By emphasizing the vital role that leadership practices play in forming nurses’ professional competencies which are necessary for negotiating the intricate and ever-changing healthcare landscape this study advances the nursing profession. The study emphasizes how adaptive leadership approaches can enable nurses to improve their abilities, resilience, and professional development by looking at the mediating role of strategic flexibility. For nursing leaders and legislators looking to establish nurturing cultures that encourage development and flexibility in their staff, eventually enhancing patient outcomes and healthcare efficiency, an understanding of this link offers important insights. Additionally, this study tackles the urgent need for modern healthcare systems to develop a workforce of skilled and flexible nurses. The study offers a framework for creating focused interventions and leadership development initiatives by tying strategic flexibility to career competencies and leadership practices. In addition to helping nurses progress in their careers, this improves organizational performance and makes healthcare systems more equipped to meet new problems. The results advance the field of healthcare administration by providing useful tactics for improving workforce competencies in a setting that is becoming more complicated and demanding.

With the standup of the nursing labor nowadays, it is regarding that information is scarce concerning the intertwined connections among nurses’ competencies, strategic flexibility, and leadership practices, underscoring the necessity of effective leadership that cultivates a flexible and encouraging work environment. By recognizing these connections, the study offers insightful information that can guide professional development and nursing education programs in Egypt, ultimately resulting in a more knowledgeable and adaptable nursing workforce [21].

The study’s findings also have wider implications for Egyptian healthcare policy and organizational practices. Supporting leadership styles that enhance strategic flexibility can help healthcare companies create an atmosphere that encourages nursing practitioners to innovate and continuously improve [20]. In Egypt, where healthcare systems are dealing with several issues, such as a growing patient population and limited resources, this is especially crucial. By putting the study’s suggestions into practice, nurses’ job satisfaction and retention rates may increase, bolstering the healthcare workforce. In the end, this study acts as a stimulant for the development of a more efficient nursing profession in Egypt, which is essential for improving health outcomes and raising the standard of healthcare services provided there generally.

### The research questions are

- What are nurses’ levels of career competencies, multifactorial leadership behavior, and strategic flexibility?
- Is there a connection between nurses’ strategic flexibility, career competencies, and multifactorial leadership behavior?
- Is there a relationship between nurses’ career competencies and multifactorial leadership behavior that mediates strategic flexibility?

## Methods

### Study design

In an Egyptian hospital, a descriptive correlational cross-sectional study design was used adhering to STROBE criteria.

### Setting

This study was conducted in El-Rahmania General Hospital, a 220-bed facility run by the Ministry of Health and Population (MOHP). This included all inpatient intensive care units (ICUs) (*n* = 16) as well as medical and surgical care units. Three surgical units (*n* = 3), five intensive care units (ICUs) (general, neonatal, pediatric, coronary care, and emergency); and eight medical units (obstetric, pediatric, neurosurgery, dialysis, orthopedic, and poison units). The selected setting comprises a diversity of departments and appraises the implementation of patient safety measures across diverse clinical contexts. This diversity allows for a comprehensive assessment of the changes. This hospital is now undergoing the process of national accreditation. This guarantees that the research is pertinent to the particular criteria and specifications established by the regulatory body.

The hospital setting was chosen for this study because of its standing as a top healthcare facility with a strong dedication to nursing quality and professional growth. Examining the connection between leadership practices and nurses’ professional competencies is made possible by the hospital’s implementation of several leadership models that emphasize strategic flexibility. Furthermore, this hospital offers a rich setting for examining how leadership affects nurses’ adaptability and professional development because it serves a varied patient population and functions in a dynamic healthcare environment. Its eligibility for this research is further supported by the hospital’s continuing workforce development and leadership training activities, which guarantee pertinent and useful insights that may be extended to larger healthcare settings.

### Sampling

Using a convenience non-probability selection technique, the study chose nurses from the target group who satisfied particular qualifying requirements. Due to practical limitations and the requirement to gather data from a sample that was easily accessible within the hospital, this strategy was selected. 400 nurses (*n* = 400) who had been employed for at least a year in the chosen hospital units made up the target group. To ensure that participants had enough experience and exposure to the workplace to offer insightful comments on the study’s emphasis on leadership practices, strategic flexibility, and career competencies, these nurses had to have direct patient care duties. Three requirements had to be met to be eligible to participate: (1) nurses had to work for at least a year in one of the chosen units, guaranteeing that participants had a firm grasp of the hospital’s procedures and policies; (2) nurses had to work in particular pre-selected hospital units that were chosen for their applicability to the study’s goals; and (3) nurses had to be actively involved in patient care, guaranteeing that their experiences were closely related to the day-to-day realities of providing healthcare. The data collection phase, which took place between September and November 2024, required the nurses to be present. With participants who had sufficient experience to critically answer the survey and consider the results, this method made sure the sample was pertinent.

### Instruments

#### Sociodemographic information questionnaire

The researchers inquired about the participant’s age, gender, nursing experience, years of service, years of experience in the current working unit, and educational background.

#### Multifactor leadership questionnaire (MLQ) for 5x-shortTool 

Rowold (2009) created this questionnaire [8]. Bedside nurses utilize the MLQ to gauge leadership behaviors in three distinct types, which are as follows: The 20 items used to measure transformational leadership behavior are broken down into five major sub-items, with four items for each of the following behaviors: intellectual stimulation, motivational inspiration, individual attention, idealized influence (attributed), and idealized influence (behavior); Eight items are used to quantify transactional leadership behavior. It is separated into two primary categories: management by exception (active) (4 things) and contingent reward (4 items). Additionally, passive/avoidant behavior is divided into two sub-items: laissez-faire and management by exception (passive).

Eight items will also be used to measure the final two categories. Each of the three aforementioned styles’ questions will be scored using a Likert scale that goes from 0 (not at all, 1 Occasionally, 2 Occasionally, 3 Quite frequently, 4 Often, if not always. It has been established that the MLQ’s validity and reliability range from 0.74 to 0.94 (Rowold, 2009) (1). The grading method was altered by the researcher. A three-point Likert scale will be created from the five-point one. This new scale will group 0 (not at all) and 1 (once in a while) into 1 “Once in a while,” 2 (sometimes) into 2 “Sometimes,” and 3 (quite often” and “often, if not always”) into 3 “frequently, if not always.” With 36–59 denoting little perceived leadership style, 60–85 denoting moderate perceived leadership style, and 86–108 denoting maximum perceived leadership style, the total score ranged from 36 to 108.

#### Career competencies scale (CCS)

Yamada et al. (2022) created this utility [14]. This is intended to gauge how nurses view careers connected to competence. The 21 items in this tool are divided into three dimensions: Seven items measure behavioral professional competencies, seven measure communicative career abilities, and seven measure reflective career competencies. Each item is rated on a five-point Likert-type scale, with 1 denoting total disagreement and 5 denoting total agreement. The findings are analyzed using the total of the scores on each subscale. The scale’s overall score falls between 21 and 105. Greater career competencies are indicated by higher ratings. Cronbach’s α scores for the 21-item CCQ-J and its subscales ranged from 0.85 to 0.95.

#### Organizational flexibility scale (OFS)

This tool was developed by Al Khalifa et al. (2021) [19]. This is used to assess nurses’ awareness of strategic flexibility. This tool consists of 17 items which contain four dimensions namely: flexibility of capacities (4 items), flexibility of resource (3 items), flexibility of information (5 items), and flexibility of coordination (5 items). Each item is rated on a five-point Likert scale, where 1 represents “strongly disagree” and 5 represents “strongly agree.” The findings are analyzed using the total of the scores on each subscale. The scale’s overall score falls between 17 and 85. Greater career competencies are indicated by higher ratings. Cronbach’s α values for the 17-item subscales ranged from 0.689 to 0.885.

### Tools validity

After being translated into Arabic and English, the three instruments underwent modifications. Seven experts were provided with resources to analyze, evaluate, and remark on item clarity, question types, and content validity. These experts included four professors and three assistant professors from the Department of Nursing Administration, at Alexandria University. Their recommendations were taken into account in order to guarantee precision and safeguard the study. To ensure accuracy, a confirmatory factor analysis was performed on the Organizational Flexibility Scale, Career Competencies Scale, and Multifactor Leadership Questionnaire. The Kaiser-Meyer-Olkin (KMO) and the Bartlett Test of Sphericity were the first instruments used to evaluate sample adequacy. For the Bartlett Test of Sphericity, a minimum KMO value of 0.60 and a significance level of 0.05 are required.

The Multifactor Leadership Questionnaire, Career Competencies Scale, and Organizational Flexibility Scale all had values of 0.931 (P 0.000), 0.918 (P 0.000), and 0.899 (P 0.000), respectively, rendering the results. All of the concepts examined in this study had factor loadings that exceeded the suggested threshold of 0.70 [22]. confirming the scales’ construct validity. The average variance extracted (AVE) values for every study variable dimension further support convergent validity. Convergent validity was assessed using each component’s average variance extracted (AVE) values [23]. AVE values greater than 0.50, which demonstrate that the construct explains the majority of the variance, are indicative of convergent validity. Discriminant validity was assessed by comparing the squared correlations between the constructs with the AVE values.

Since each AVE value was above the squared correlations, discriminant validity was deemed to be satisfied. As a result, the measures employed in this investigation were found to possess both discriminant and convergent validity.

### Ethical considerations

The study’s approach was approved by the College of Nursing’s Damanhour University Research Ethics Committee (Research code: 103 n). Before signing their consent, nurses were briefed on the study’s objectives. To ensure confidentiality and identity protection, each questionnaire was assigned a code number. Data would only be used for study, nurses were informed. The ability to leave the study has been confirmed.

### Pilot study and reliability

To maintain the products’ practicality and straightforwardness and to see any potential problems during data collection, 10% of the nurses from the same hospital (*n* = 40) gave their approval for the pilot project. There was nothing to be altered. Participants in the pilot trial were not included in the study to avoid data contamination. The researchers reviewed the surveys to ensure they were inclusive and accurate. To ascertain the reliability of research instruments, the internal consistency of the items was measured with Cronbach’s alpha coefficient test.

### Data quality control techniques

The study used several data quality control methods to guarantee the authenticity and dependability of the data. First, information on nurses’ career competencies, strategic flexibility, and leadership practices was gathered using standardized and verified measurement tools. To guarantee their accuracy and usefulness in capturing the factors of interest, these instruments were carefully chosen based on previous research. To make sure that participants understood the survey or interview questions completely, pilot testing was done before full-scale data collecting to find and fix any misunderstandings. This procedure improved the data-gathering tools’ clarity and reduced the possibility of answer errors.

Second, to preserve data integrity, the study put procedural controls in place. To lessen social desirability bias and promote truthful responses, participants were guaranteed anonymity and confidentiality. To further guarantee uniformity and respect to established procedures, the data collection method was assisted by qualified researchers. As soon as the data was gathered, it was checked for accuracy and completeness, and any missing or inconsistent responses were dealt with right away. During the analysis stage, statistical methods like data normalization and outlier detection were used to make sure that abnormalities or mistakes in data entry wouldn’t skew the results. Together, these stringent quality control procedures enhanced the validity of the study’s conclusions.

### Data collection

To minimize potential biases and guarantee the accuracy and completeness of the responses, the data-gathering procedure was created. The researchers sent each nurse a hard copy questionnaire so that they could personally discuss the goal of the study. The researcher gave a two-minute description of the study before it started, stressing its significance and reassuring participants of their anonymity and secrecy. This strategy sought to build confidence and promote open communication. To make sure they understood the topics being covered, nurses were given the chance to clarify anything they didn’t understand or ask questions before filling out the questionnaire.

The questionnaires were filled out in front of the researcher to improve data quality. This reduced the possibility of incomplete or missing responses and guaranteed that all questions were addressed. When participants had questions or concerns during the completion procedure, the researchers answered them right away. Nurses completed the questionnaire in an average of 15 to 20 min, striking a balance between efficiency and completeness to fit their hectic schedules. Two months, from September 2024 to November 2024, were allotted for data collection, which gave enough time to interact with individuals and obtain a representative sample. Accurate, trustworthy, and thorough data gathering for the study was guaranteed by this methodical and participatory methodology.

### Data analysis

IBM SPSS Statistics (Version 23) and IBM SPSS AMOS (Version 23) were used to analyze the data. Frequencies and percentages were utilized to describe the individuals’ demographic characteristics. Using means and standard deviations, the three primary study variables leadership practices, strategic flexibility, and nursing career competencies were explained. A one-way analysis of variance and an independent sample t-test was used to determine whether the research variable varied depending on demographic factors.

Pearson’s correlation analysis was used to determine the link between the main research variables. To determine whether there was a direct correlation between leadership practices and nursing career abilities, regression analyses were employed. A structural equation model was used to examine the indirect impact of strategic flexibility on the connection between leadership practices and nurses’ career capacities. The study employed Cronbach’s alpha and composite reliability (CR) to verify the validity of the scale items. The accuracy of the study’s components was further ensured by conducting multiple confirmatory factor analyses.

## Results

Table 1 revealed the distribution of 400 studied nurses rendering demographic data. The largest group, with a mean age of 39.23 ± 11.45 years, is those over 40 (34.8%), followed by those under 50 (24.0%). Males represent only 19.3% of the sample; while the females constitute the majority, 80.8%. Moreover, the majority of nurses (70.5%) are single, with married nurses falling in second (22.8%). Regarding qualification, 65.0% are technical nurses, while just 12.5% are professional nurses. Also, pediatric nurses make up the largest group (40.3%). additionally, 33.8% had less than 5 years’ experience, with a mean of 8.62 ± 6.75 years of experience in nursing. Moreover, the majority of nurses (51.3%) have worked in a hospital for one to five years, an average of 5.89 ± 5.02 years. in addition, The vast majority (77.3%) have gone to leadership training sessions.

**Table 1:** Distribution of the studied nurses according to demographic data (*n* = 400)

Demographic characteristics	No.	%
**Age (years)**		
20–	94	23.5
30–	71	17.8
40–	139	34.8
≥ 50	96	24.0
Mean ± SD	39.23 ± 11.45	
**Gender**		
Male	77	19.3
Female	323	80.8
**Marital status**		
Married	91	22.8
Single	282	70.5
Widowed	18	4.5
Divorced	9	2.3
**Educational Qualification**		
Bachelor science in nursing	50	12.5
Technical Nursing Institute	260	65.0
Nursing School Diploma	90	22.5
**Job position**		
Word nurse	34	8.5
ICU nurse	64	16.0
CCU nurse	42	10.5
Pediatric nurse	161	40.3
OR nurse	56	14.0
Emergency nurse	39	9.8
Others	4	1.0
**Experience year of nursing**		
< 5	135	33.8
5–10	72	18.0
10–15	122	30.5
15 - <20	44	11.0
≥ 20	27	6.8
Mean ± SD	8.62 ± 6.75	
**Experience hospital**		
< 1	12	3.0
1–5	205	51.3
5–10	64	16.0
10–15	78	19.5
15 - <20	17	4.3
≥ 20	24	6.0
Mean ± SD	5.89 ± 5.02	
**Are you attend any training course**		
Yes	309	77.3
No	91	22.8

### Perceived level of multifactorial leadership behaviors, career competencies, and organizational flexibility among nurses

Table 2 shows the distribution of the studied nurses regarding the level of perception related to multifactorial leadership behaviors, career competencies, and organizational flexibility. regarding multifactorial leadership behaviors, 53.5% of nurses had a moderate perception of multifactorial leadership with a total mean score of 70.74 ± 17.29. in the transformational leadership behavior dimension, the majority of nurses (61.5%) had idealized influence attributes, with a mean percent score of 69.53% and a mean score of 9.56 ± 2.66 which indicates the majority of nurses view leaders as strong, moral role models. on the other hand, only 14.8% of the nurses had a high score in inspirational motivation which suggests that a large number of nurses might not be sufficiently inspired or motivated by the vision of their leaders, which could affect their feeling of engagement or purpose.

**Table 2:** Distribution of the studied nurses according to their levels and mean percent score (*n* = 400)

	Low (< 33.3%)		Moderate (33.3– <66.6%)		High (≥ 66.6%)		Total score	Mean score	Mean percent score
	No.	%	No.	%	No.	%	Mean ± SD	Mean ± SD	Mean ± SD
**Transformational Leadership Behaviors**									
Idealized influence Attribute	51	12.8	103	25.8	246	61.5	9.56 ± 2.66	2.39 ± 0.67	69.53 + 33.30
Idealized influence Behavior	87	21.8	187	46.8	126	31.5	8.49 ± 2.11	2.12 ± 0.53	56.13 ± 26.40
Inspirational motivation	166	41.5	175	43.8	59	14.8	7.28 ± 2.46	1.82 ± 0.61	40.94 ± 30.69
Intellectual stimulation	15	3.8	178	44.5	207	51.8	9.47 ± 2.07	2.37 ± 0.52	68.38 ± 25.92
Individual Consideration	300	75.0	37	9.3	63	15.8	5.63 ± 2.99	1.41 ± 0.75	20.38 ± 37.34
**Overall Transformational Leadership Behaviors**	**53**	**13.3**	**286**	**71.5**	**61**	**15.2**	**40.43 ± 9.16**	**2.02 ± 0.46**	**51.07 ± 22.90**
**Transactional Leadership Behavior**									
Contingent Reward	187	46.8	79	19.8	134	33.5	7.48 ± 3.22	1.87 ± 0.81	43.44 ± 40.31
Management by Exception (Active)	121	30.3	106	26.5	173	43.3	9.06 ± 2.73	2.26 ± 0.68	63.19 ± 34.13
**Overall Transactional Leadership Behavior**	**159**	**39.7**	**71**	**17.8**	**170**	**42.5**	**16.53 ± 5.05**	**2.07 ± 0.63**	**53.31 ± 31.58**
**Passive/avoidant behavior**									
Management by Exception (Passive)	49	12.3	212	53.0	139	34.8	8.42 ± 2.26	2.11 ± 0.56	55.28 ± 28.23
Laissez-Faire	332	83.0	0	0.0	68	17.0	5.36 ± 3.01	1.34 ± 0.75	17.0 ± 37.61
**Overall Passive/avoidant behavior**	**260**	**65.0**	**77**	**19.2**	**63**	**15.7**	**13.78 ± 4.82**	**1.72 ± 0.60**	**36.14 ± 30.12**
**Overall Multifactor Leadership Questionnaire (MLQ)**	**118**	**29.5**	**214**	**53.5**	**68**	**17.0**	**70.74 ± 17.29**	**1.70 ± 0.65**	**36.02 ± 12.42**
Flexibility of Capacities	9	2.3	131	32.8	260	65.0	16.17 ± 3.60	4.04 ± 0.90	76.03 ± 22.50
Flexibility of Resource	152	38.0	136	34.0	112	28.0	9.13 ± 3.14	3.05 ± 1.05	51.13 ± 26.16
Flexibility of Information	198	49.5	39	9.8	163	40.8	15.16 ± 7.85	3.03 ± 1.57	50.77 ± 39.27
Flexibility of Coordination	45	11.3	101	25.3	254	63.5	18.28 ± 4.28	3.66 ± 0.86	66.39 ± 21.40
**Overall Strategic Flexibility**	**36**	**9.0**	**201**	**50.3**	**163**	**40.8**	**58.73 ± 13.84**	**3.44 ± 0.78**	**61.08 ± 19.57**
Reflective career competences	5	1.3	0	0.0	395	98.8	28.77 ± 2.97	4.11 ± 0.42	77.75 ± 10.61
Communicative career competences	7	1.8	104	26.0	289	72.3	26.47 ± 5.99	3.78 ± 0.86	69.54 ± 21.40
Behavioral career competences	10	2.5	110	27.5	280	70.0	27.44 ± 5.35	3.92 ± 0.76	72.98 ± 19.12
**Overall Career Competencies Scale**	**6**	**1.5**	**105**	**26.3**	**289**	**72.3**	**82.68 ± 12.87**	**3.94 ± 0.61**	**73.43 ± 15.32**

SD: Standard deviation

furthermore, regarding transactional leadership behavior, and contingent reward ratings, just 33.5% of nurses believe that they are highly motivated by rewards. however, 43.3% of nurses believe that management by exception (active) is high, indicating that leaders frequently step in to fix errors. Although this emphasizes a problem-oriented strategy, it could come out as reactive leadership as opposed to proactive leadership.

Moreover, in passive/avoidant leadership behaviors, nurses’ perception is moderate level 53% with a mean score of 8.42 ± 2.26 for management by exception (passive) indicating that some leaders may wait to act until issues get serious before taking action. furthermore, 83% of nurses received a poor laissez-faire score with a mean score of 5.36 ± 3.01. Moreover, Table 2 demonstrated that in terms of strategic flexibility around half of the nurses had a moderate perception of strategic flexibility with a total mean score of 58.73 ± 13.84. Furthermore, the majority of nurses worked exceptionally well in terms of flexibility of capacities (65% high scores), with a total mean score of 16.17 ± 3.60 indicating adaptability.

Conversely, nurses’ total mean percent scores fluctuated around 50%, indicating difficulties with resource and information management. Moreover, according to career competencies, Table 2 reveals most nurses 72.3% had a high perception of career competencies with a total mean score of 82.68 ± 12.87. in reflective career competencies, dimension had a mean percent score of 77.75% and a maximum score of 98.8%, allowing them to stand out. This implies that nurses think very critically about their careers. A large majority of nurses (more than 70%) received high scores in the areas of behavioral and communicative competencies.

### Relationship between multifactorial leadership behaviors, career competencies, and organizational flexibility among nurses

Table 3 shows the correlation between multifactorial leadership behaviors, strategic flexibility, and career competencies. this table revealed overall transformational leadership has strong positive correlations with overall transactional leadership (*r* = 0.602, *p* < 0.001). additionally, the higher correlation of overall transformational leadership with overall passive/avoidant leadership (0.866). furthermore, overall strategic flexibility has a weak positive statistically significant difference (*r* = 0.111, *p* < 0.026) with overall multifactorial leadership and also, a weak positive statistically significant correlation (*r* = 0.171, *p* < 0.001) with overall career competencies. furthermore, weak positive statistically significant correlation between multifactorial leadership behavior and career competencies (*r* = 0.189, *p* < 0.001).

**Table 3:** Correlation between Multifactor Leadership Practices, Strategic Flexibility, and Career Competencies among nurses (*n* = 400)

		Attribute	Behavior	Inspirational motivation	Intellectual stimulation	Individual Consideration	Overall Transformational Leadership Behaviors	Contingent Reward	Management by Exception (Active)	Overall Transactional Leadership Behavior	Management by Exception (Passive)	Laissez-Faire	Overall Passive/avoidant behavior	Overall Multifactor Leadership ership Behaviors	Flexibility of Capacities	Flexibility of Resource	Flexibility of Information	Flexibility of Coordination	Overall Strategic Flexibility	Reflective career competences	Communicative career competences	Behavioral career competences	Overall Career Competencies Scale
Attribute	**r**																						
	**p**																						
Behavior	**r**	0.522*																					
	**p**	< 0.001*																					
Inspirational motivation	**r**	0.047	0.379*																				
	**p**	0.348	< 0.001*																				
Intellectual stimulation	**r**	0.878*	0.702*	0.050																			
	**p**	< 0.001*	< 0.001*	0.315																			
Individual Consideration	**r**	0.364*	0.458*	0.730*	0.421*																		
	**p**	< 0.001*	< 0.001*	< 0.001*	< 0.001*																		
**Overall Transformational Leadership Behaviors**	**r**	0.716*	0.793*	0.591*	0.795*	0.829*																	
	**p**	< 0.001*	< 0.001*	< 0.001*	< 0.001*	< 0.001*																	
Contingent Reward	**r**	0.275*	0.445*	0.273*	0.361*	0.459*	0.487*																
	**p**	< 0.001*	< 0.001*	< 0.001*	< 0.001*	< 0.001*	< 0.001*																
Management by Exception (Active)	**r**	0.501*	0.096	0.336*	0.443*	0.570*	0.545*	0.435*															
	**p**	< 0.001*	0.055	< 0.001*	< 0.001*	< 0.001*	< 0.001*	< 0.001*															
**Overall Transactional Leadership Behavior**	**r**	0.447*	0.336*	0.356*	0.470*	0.602*	0.605*	0.874*	0.818*														
	**p**	< 0.001*	< 0.001*	< 0.001*	< 0.001*	< 0.001*	< 0.001*	< 0.001*	< 0.001*														
Management by Exception (Passive)	**r**	0.651*	0.613*	0.446*	0.569*	0.727*	0.816*	0.578*	0.527*	0.654*													
	**p**	< 0.001*	< 0.001*	< 0.001*	< 0.001*	< 0.001*	< 0.001*	< 0.001*	< 0.001*	< 0.001*													
Laissez-Faire	**r**	0.320*	0.643*	0.693*	0.463*	0.841*	0.806*	0.545*	0.396*	0.562*	0.668*												
	**p**	< 0.001*	< 0.001*	< 0.001*	< 0.001*	< 0.001*	< 0.001*	< 0.001*	< 0.001*	< 0.001*	< 0.001*												
**Overall Passive/avoidant behavior**	**r**	0.505*	0.689*	0.642*	0.556*	0.866*	0.886*	0.611*	0.494*	0.657*	0.885*	0.937*											
	**p**	< 0.001*	< 0.001*	< 0.001*	< 0.001*	< 0.001*	< 0.001*	< 0.001*	< 0.001*	< 0.001*	< 0.001*	< 0.001*											
**Overall Multifactor Leadership**	**r**	0.651*	0.710*	0.596*	0.713*	0.856*	0.206*	0.684*	0.665*	0.260*	0.870*	0.853*	0.207*										
	**p**	< 0.001*	< 0.001*	< 0.001*	< 0.001*	< 0.001*	< 0.001*	< 0.001*	< 0.001*	0.000	< 0.001*	< 0.001*	< 0.001*										
Flexibility of Capacities	**r**	0.187*	0.005	0.130*	0.172*	0.072*	0.034	0.082	0.036	0.033	0.027	0.064	0.027	0.001									
	**p**	< 0.001*	0.928	0.009*	0.001*	0.151*	0.500	0.103	0.471	0.515	0.589	0.199	0.584	0.989									
Flexibility of Resource	**r**	0.093	0.022	0.046	0.080	0.172*	0.119*	-0.027	0.055	0.012	0.034	0.235*	0.163*	0.112*	0.060								
	**p**	0.063	0.664	0.363	0.109	0.001*	0.018*	0.588	0.275	0.807	0.502	0.000*	0.001*	0.025*	0.229								
Flexibility of Information	**r**	0.238*	0.007	0.109*	0.272*	0.103*	0.134*	0.063	0.065	0.005	0.060	0.189*	0.090	0.094	0.212*	0.682*							
	**p**	< 0.001*	0.896	0.029*	< 0.001*	0.039*	0.007*	0.210	0.197	0.917	0.230	< 0.001*	0.073	0.060	< 0.001*	< 0.001*							
Flexibility of Coordination	**r**	0.197*	0.032	0.138*	0.228*	0.096	0.111*	0.064	0.068	0.078	0.031	0.157*	0.084	0.105*	0.296*	0.466*	0.697*						
	**p**	< 0.001*	0.526	0.006*	< 0.001*	0.054	0.027*	0.202	0.172	0.120	0.542	0.002*	0.094	0.036*	< 0.001*	< 0.001*	< 0.001*						
**Overall Strategic Flexibility**	**r**	0.168*	0.012	0.061	0.199*	0.146*	0.127*	0.001	0.061	0.032	0.043	0.226*	0.130*	0.111*	0.218*	0.742*	0.882*	0.887*					
	**p**	0.001*	0.808	0.226	< 0.001*	0.003*	0.011*	0.987	0.225	0.522	0.391	< 0.001*	0.009*	0.026*	< 0.001*	< 0.001*	< 0.001*	< 0.001*					
Reflective career competences	**r**	0.056	0.128*	0.178*	0.072	0.236*	0.187*	0.074	0.054	0.076	0.136*	0.286*	0.243*	0.189*	0.027	0.255*	0.212*	0.308*	0.281*				
	**p**	0.261	0.010*	< 0.001*	0.152	< 0.001*	< 0.001*	0.141	0.280	0.128	0.006*	< 0.001*	< 0.001*	< 0.001*	0.587	< 0.001*	< 0.001*	< 0.001*	< 0.001*				
Communicative career competences	**r**	0.267*	0.325*	0.082	0.269*	0.067	0.213*	0.074	0.043	0.024	0.117*	0.202*	0.181*	0.170*	0.146*	0.249*	0.221*	0.229*	0.215*	0.652*			
	**p**	< 0.001*	< 0.001*	0.100	< 0.001*	0.183	< 0.001*	0.139	0.393	0.630	0.020*	< 0.001*	< 0.001*	0.001*	0.003*	< 0.001*	< 0.001*	< 0.001*	< 0.001*	< 0.001*			
Behavioral career competences	**r**	0.204*	0.343*	0.024	0.166*	0.041	0.183*	0.116	0.098	0.021	0.186*	0.185*	0.202*	0.160*	0.127*	0.150*	0.025	0.000	0.016	0.480*	0.839*		
	**p**	< 0.001*	< 0.001*	0.630	0.001*	0.409	< 0.001*	0.021	0.051	0.677	< 0.001*	< 0.001*	< 0.001*	0.001*	0.011*	0.003*	0.612	0.995	0.756	< 0.001*	< 0.001*		
**Overall Career Competencies Scale**	**r**	0.222*	0.324*	0.007	0.210*	0.103*	0.218*	0.100*	0.048	0.038	0.163*	0.237*	0.224*	0.189*	0.114*	0.237*	0.163*	0.178*	0.171*	0.734*	0.965*	0.917*	
	**p**	< 0.001*	< 0.001*	0.882	< 0.001*	0.040*	< 0.001*	0.046*	0.336	0.454	0.001*	< 0.001*	< 0.001*	< 0.001*	0.022*	< 0.001*	0.001*	< 0.001*	0.001*	< 0.001*	< 0.001*	< 0.001*	

r: Pearson coefficient *: Statistically significant at *p* ≤ 0.05

The findings in Table 4 show that both strategic flexibility and transformational leadership behaviors are statistically significant predictors in the model. With a beta value of 0.128, a t-value of 3.518, and a *p*-value below 0.001, Transformational Leadership Behaviors appear to have a significant and positive correlation with the dependent variable. The positive and significant influence of Strategic Flexibility is also confirmed by its beta of 0.142, t-value of 3.106, and *p*-value of 0.002. The relevance of both variables is further supported by the fact that their 95% CIs do not contain zero. With an adjusted R2 of 0.054 and a 6% variance explanation (R2 = 0.060), the model is significant overall (F = 12.377, *p* < 0.001), indicating that the predictors taken together offer a significant explanation for the variation in the result.

**Table 4:** Multivariate Linear Regression Analysis for Factors Affecting Career Competencies (*n* = 400)

Variable	B	Beta	t	*p*	95% CI	
					LL	UL
Transformational Leadership Behaviors	0.128	0.172	3.518*	< 0.001*	0.057	0.200
**Strategic Flexibility**	**0.142**	**0.152**	3.106*	0.002*	**0.052**	**0.231**
**R**^**2**^ **= 0.060**,** Adjusted R**^**2**^ **= 0.054**, F = 12.377^*^, *p* < 0.001^*^						

F, p: f and *p* values for the model.

R^2^: Coefficient of determination.

B: Unstandardized Coefficients.

Beta: Standardized Coefficients.

t: t-test of significance.

LL: Lower limit UL: Upper Limit.

*: Statistically significant at *p* ≤ 0.05

### Standardized regression coefficient weights among leadership behaviors, career competencies, and strategic flexibility with the mediating role of strategic flexibility

**Fig. 2:**
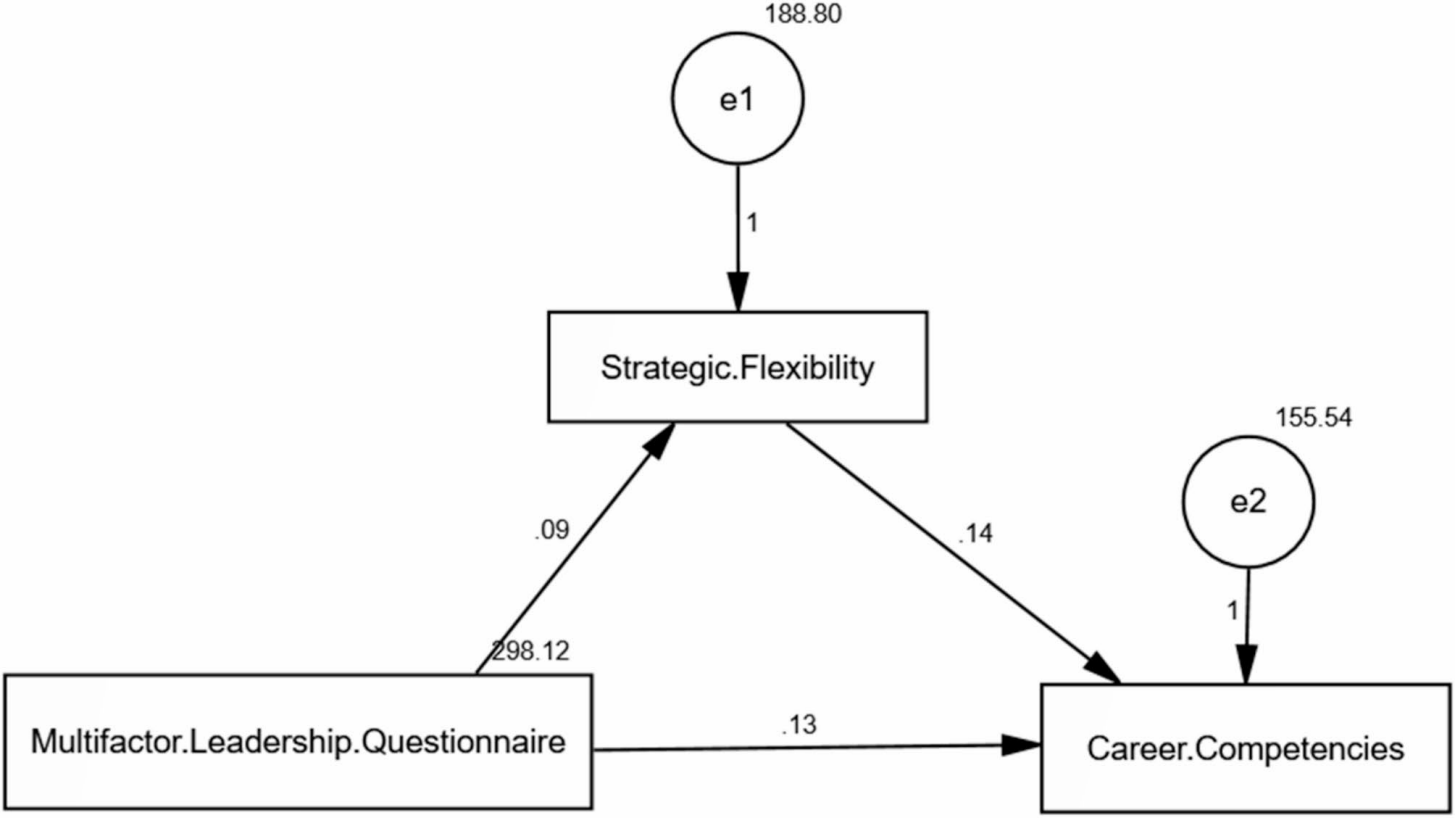
Path analysis of the direct and indirect effect of Multifactorial Leadership Behaviors on Career Competencies mediating by Strategic Flexibility among nurses

Table 5 examines the direct and indirect effects of leadership behaviors on career competencies through the mediating factor of strategic flexibility. The study reveals a significant direct effect (Estimate = 0.089, *p* = 0.026) between leadership behaviors and strategic flexibility as also revealed in Fig. 2. This suggests that strategic flexibility is positively influenced by leadership. Moreover, there is a significant direct influence of leadership behaviors on career competencies (Estimate = 0.128, *p* < 0.001), indicating that effective leadership directly enhances career competencies. Furthermore, A significant indirect effect (Estimate = 0.142, *p* = 0.002) is found in the relationship between strategic flexibility and career competencies, indicating that enhancing strategic flexibility enhances the impact of leadership on career competencies. Entirely of these indicators refer to a good representation of the observed data through the model (Table 4 and Fig. 2).

**Table 5:** The direct and indirect effect of Multifactorial Leadership Behaviors on nurses’ Career Competencies as mediated by Strategic Flexibility

			Direct effect	Indirect effect	Estimate	S.E.	C.*R*.	*P*
**Strategic Flexibility**	<---	**Multifactorial Leadership Behaviors**	0.089		0.111	0.04	2.232*	0.026*
**Career Competencies**	<---	**Multifactorial Leadership Behaviors**	0.128	0.013	0.172	0.036	3.526*	< 0.001*
**Career Competencies**	<---	**Strategic Flexibility**	0.142		0.152	0.045	3.114*	0.002*

Model fit parameters CFI; IFI; RMSEA (0.951; 0.902; 0.094).

Model χ^2^/df. 9.695/3 *p* ≤ 0.001.

CFI: Comparative Fit Index, IFI: Incremental Fit Index, RMSEA: Root Mean Square Error of Approximation

## Discussion

Effective leadership encourages flexibility, which improves critical thinking, communication, and problem-solving abilities, all of which are crucial for nursing capabilities. Nurses report higher levels of readiness and job satisfaction as hospitals adopt adaptable, responsive leadership styles, which can eventually enhance patient care quality [24, 25]. Enhancing strategic flexibility increases the influence of leadership on career growth, according to this study, which also identified a substantial indirect connection between strategic flexibility and competence.

This study demonstrates a strong direct correlation between leadership characteristics and strategic flexibility. Additionally, a significant direct relationship between vocational competencies and leadership behaviors has been noted. There is a strong correlation between leadership behaviors and career abilities since these practices have a big influence on nurses’ professional growth and advancement. Effective leadership behaviors, such as providing guidance, fostering a supportive environment, and promoting continuous learning, have a direct impact on nurses’ capacity to build and enhance their competencies. Nurses may think critically, take on new challenges, and acquire critical thinking skills all of which are vital for career advancement—when their supervisors use participative or transformational leadership styles.

Furthermore, leadership practices that prioritize professional growth, mentoring, and acknowledgment boost nurses’ self-esteem and competence, enabling them to handle challenging patient care scenarios and advance in their careers. This link demonstrates how leadership is essential to developing nursing professionals’ competencies as well as influencing organizational learning. These results were compatible with Hensellek et al. (2023) leadership was significantly and positively related to strategic flexibility [26]. Additionally, Sanders (2024) showed that leaders used adaptable leadership, performance-based monitoring, high-quality relationships, and intentional communication. These actions were effective tactics that supported career growth [27].

Furthermore, this study found that nurses had moderate opinions about multidimensional leadership. In the transformational leadership behavior dimension, most nurses had the idealized influence trait. These results could be explained by the fact that nurses frequently operate in high-stress settings where good cooperation and patient care depend on excellent leadership. Although nurses acknowledge the existence of diverse leadership styles, their moderate views regarding multidimensional leadership may suggest that they do not view them as consistently strong or weak across all dimensions. According to the idealized influence trait’s preponderance in transformational leadership, nurse leaders act as role models for their teams by acting morally, being dedicated, and having a clear vision. This quality is consistent with the core values of the nursing profession, which include integrity, trust, and patient-centered care. However, because of institutional limitations, workload demands, or differences in leadership training, some elements of transformational leadership, such as personalized attention or intellectual stimulation, could be less noticeable. While acknowledging the substantial role of idealized influence in forming nursing practice, these elements might also be responsible for the moderate overall view of multidimensional leadership.

This study is compatible with Thanh and Quang (2022) who revealed a mean score of 3.88 and a standard deviation of 0.386 indicating that transformational leadership was the most frequently encountered leadership style [28].

Moreover, nurses’ moderate degree of impression of management by exception (passive) suggests that some leaders would put off taking action until problems become more serious. Pay close attention to anomalies, errors, expectations, and departures from norms. records all errors and focuses my attention on instances when standards aren’t being met. On the other hand, 83% of nurses reported receiving weak, laissez-faire leadership; this could be because nurses took charge of making decisions, promptly addressed pressing inquiries, and intervened when significant difficulties arose. These results are contradicted by Mohamed et al. (2022) showed more of the first-line nurse managers (53.6%) had a high level of laissez-faire style [29].

Furthermore, about one-half of the nurses indicated that their strategic flexibility was modest. Most nurses performed remarkably well in terms of capacity flexibility. These findings could be explained by the fact that, although nurses understand the importance of flexibility in their work, several organizational and systemic constraints may restrict their total strategic flexibility. Rigid institutional policies, staffing shortages, or limited decision-making autonomy may be the cause of the small amount of strategic flexibility stated by roughly 50% of nurses, which may limit their capacity to react proactively to changes in healthcare environments. Despite these limitations, the high performance in capacity flexibility indicates that nurses are excellent at multitasking, modifying their workload, and effectively managing patient care. This might be explained by their practical experience, clinical training, and capacity for high-pressure work. Nurses’ capacity flexibility is increased by the frequent need to adjust to changing patient needs, emergencies, and technology improvements.

These results were agreed with Mahmoud and Amer (2024) who revealed that 75.0% of first-line nurse managers and 56.3% of staff nurses had a high level of strategic flexibility [26]. Finally, nurse managers have a high potential for using the technology that is currently available to enhance and expand their services [20]. Conversely, nurses’ overall mean percent ratings varied about 50%, suggesting information and resource challenges. Furthermore, According to this research, most nurses had a positive opinion of their career competencies, particularly in the reflective career competencies dimension. This is because nurses are aware of their strengths, talents, and areas of interest in their profession. This result is compatible with Ali et al. (2022) revealed that nurses’ perceived competency level and focus on clinical nursing competency achievement and improvement [30].

### Strengths and limitations

By filling a crucial knowledge gap on the connection between career abilities, strategic flexibility, and leadership practices in the nursing field, this study has many strong points. Its emphasis on the mediating function of strategic flexibility offers a sophisticated viewpoint, going beyond conventional leadership research to investigate how workforce growth in dynamic healthcare environments might be fueled by leadership adaptability. The study is also based in a hospital setting, providing useful and applicable insights that may be immediately implemented to enhance organizational results and leadership tactics. The reliability and generalizability of the results across comparable healthcare institutions are guaranteed by the use of strong research techniques, such as representative sampling and validated instruments.

It is important to recognize the study’s limitations despite its positives. Its dependence on data from a single hospital environment is one of its limitations, which can limit the findings’ applicability to other healthcare facilities with different organizational structures, cultural norms, or resource availability. Furthermore, the study’s cross-sectional methodology makes it more difficult to prove a link between professional competencies, strategic flexibility, and leadership practices. A deeper understanding of how these associations change over time may be possible with longitudinal research. Last but not least, participant response bias for example, nurses’ reluctance to question leadership practices—may affect the data’s accuracy, highlighting the necessity of cross-referencing results using additional qualitative or observational techniques.

## Conclusion

Since nursing is a profession known for providing compassionate care (28), developing nurses’ leadership skills is essential to advancing their professional competencies (29). The statistically significant connections among strategic flexibility, nursing career competencies, and leadership behaviors demonstrate their interdependence. The results suggest that the relationship between competent nursing practice and effective leadership may be significantly mediated by strategic flexibility. This highlights the importance of adaptable leadership styles that stand in an environment that inspires lifelong learning and professional growth, ultimately producing more qualified nurses who can meet the demands of modern healthcare.

By highlighting the mediating role of strategic flexibility between leadership practices and nurses’ career competencies, this cross-sectional study inclusively increases our thought of the relationships among these three variables. This interdependence emphasizes how important these components are to supporting nurses’ future well-being and performance in the workplace.

### Implications in nursing practice

Leadership practices have a significant impact on the development of nurses’ professional competencies, which are essential for enhancing performance and adaptability in the rapidly evolving healthcare environment. Nursing leaders should prioritize putting into practice supportive and participatory leadership styles that empower nurses and encourage the growth of their skills, critical thinking, and problem-solving abilities. By fostering an environment of ongoing learning and providing opportunities for professional growth, leaders may ensure that nurses are better equipped to meet patient care demands, handle complex healthcare challenges, and develop in their careers. Strategic flexibility serves as a mediator, highlighting the importance of adaptability in leadership styles and ensuring that protocols meet the evolving needs of healthcare settings and individual career pathways.

### Implications for nursing education and policy

This study emphasizes the necessity of including leadership development and strategic flexibility training in the nursing curriculum. Nursing education programs should place a high premium on developing competencies that support effective leadership practices and preparing future nurses to work in dynamic healthcare environments. Additionally, healthcare administrators should support mentorship programs and opportunities for ongoing professional development that emphasize flexible leadership skills for practicing nurses. Through the establishment of a structured framework that encourages strategic thinking and leadership growth, nursing education and policy can improve nurses’ overall competency. In the end, this will strengthen the healthcare system and enhance patient care [31, 32].

## Data Availability

Upon a reasonable request, the relevant author will make the datasets created and examined during the current work available.
